# Impact of crystalline orientation on Cu–Cu solid-state bonding behavior by molecular dynamics simulations

**DOI:** 10.1038/s41598-023-50427-3

**Published:** 2023-12-27

**Authors:** Hiroaki Tatsumi, C. R. Kao, Hiroshi Nishikawa

**Affiliations:** 1 Joining and Welding Research Institute, Osaka University, 11-1 Mihogaoka, Ibaraki, Osaka 567-0047 Japan; 2 Department of Materials Science and Engineering, National Taiwan University, No. 1, Sec. 4, Roosevelt Road, Taipei, 10617 Taiwan

**Keywords:** Surfaces, interfaces and thin films, Atomistic models, Metals and alloys

## Abstract

High-density electronics are hindered by the constraints of Sn-based solder joints, necessitating the exploration of Cu–Cu solid-state bonding. However, current bonding methods are expensive and time-consuming; therefore, understanding the Cu–Cu bonding mechanism is crucial for optimization. This study utilizes molecular dynamics (MD) simulation to elucidate the Cu–Cu solid-state bonding behavior, focusing on interfacial densification and diffusion phenomena. Furthermore, it highlights the influence of crystal orientation on the interfacial bonding behavior. To analyze the impact of crystal orientation, monocrystalline Cu slabs with a simplified periodic surface structure were employed to replicate surface roughness and subsequently bonded at a specific temperature. The results indicate the critical influence of crystalline orientations on the bonding process: identical orientations result in slower densification at the interface, whereas misoriented orientations significantly accelerate it. This effect, attributed to the grain boundary (GB) structures formed owing to misorientation, suggests a central role for GB diffusion in bonding progression. Diffusion coefficients calculated using the mean square displacement (MSD) confirmed these findings and exhibited significantly larger values for misoriented joints. Additionally, the simulations reveal an activation energy for GB diffusion that is lower than conventional values, highlighting the impact of the crystallographic orientation and voids at the bonding interface. Our research elucidates the role of crystalline orientation in diffusion phenomena at bonding interfaces, offering valuable implications for optimizing bonding-based manufacturing processes.

## Introduction

In recent years, the rapid proliferation of highly integrated electronics and increased electrical current density has generated a growing demand for improved bonding techniques. Sn-based solder joints commonly utilized for electronic device bonding are rapidly approaching their theoretical limitations, particularly in the latest three-dimensional integrated circuits (3D-ICs) where the input/output pitch is expected to decrease to 1 μm^1^. Conventional Sn-based solder bumps with a minimum pitch of 20 μm^2^ are prone to electrical short-circuit failures owing to intermicrobump contact during bonding. As the size of solder bumps decreases, joint characteristics are increasingly affected by the formation of Kirkendall voids and brittle intermetallic compounds (IMCs) at the interface^3^. Moreover, the reliability of solder joints is limited by the achievable current density resulting from electromigration^4^. An alternative approach is transient liquid phase (TLP) bonding, commonly referred to as solid–liquid interdiffusion (SLID) bonding^5^. High-melting-point materials such as Cu and low-melting-point materials such as Sn are supplied as layers that solidify isothermally during the bonding process, enabling a higher melting-point joint consisting of IMCs. Some studies have successfully fabricated Cu–Sn IMC joints using the TLP bonding technique with Cu/Sn microbumps and demonstrated its versatility for 3D-ICs and micro-electro-mechanical system (MEMS) packaging^6,7^. However, TLP-bonded joints still face issues, including not only low electrical and thermal conductivity but also poor mechanical properties derived from the IMCs.

Owing to their high electrical conductivity, high electromigration resistivity, excellent mechanical properties, and the absence of concerns regarding Kirkendall voiding, Cu–Cu solid-state bonding technologies have attracted considerable attention to accommodate the demands of high-end 3D-ICs^5,8–11^. This method can be employed using room-temperature bonding^12^ or a combination of room-temperature bonding and post-annealing^8,9^. Achieving activated atomically flat surfaces is crucial for optimal bonding contact and can be obtained using chemical mechanical polishing (CMP) and complex surface activation processes. However, this approach is expensive and time-consuming. An alternative method is the thermal compression process^10,11^, typically involving a bonding time of ~ 1 h at temperatures of 300–400 °C, with high contact pressure applied^5^. However, these low-throughput processes pose limitations for industrial applications.

To improve the bondability at lower temperatures and for shorter times, understanding the bonding mechanism of Cu solid-state bonding is essential. First, the removal of impurities and oxides from the bonding surfaces is crucial. Several surface-modification techniques have been proposed, including high-vacuum plasma^13^, reacting gases^14^, passivation coatings^15^, electromagnetic irradiation^16^, and Ag thin layer deposition^17^. After successfully removing oxides and impurities from the bonding surfaces, the key step is to eliminate gaps at the bonded interface via deformation and diffusion. The process of traditional solid-state bonding, known as diffusion bonding, has been theoretically explained in previous literature^18,19^. The significance of interfacial plastic deformation, creep deformation, and surface/grain boundary (GB) diffusion in the formation of neck surfaces has been highlighted. Higher bonding temperatures, pressures, and times are known to improve bondability. For semiconductor applications requiring bonding under relatively low-temperature and low-pressure conditions, diffusion phenomena have a significant impact^20,21^. One dominant mechanism driving Cu–Cu solid-state bonding is GB diffusion. Rebhan and Hinger^22^ systematically evaluated the effects of possible dominant parameters on bondability by comparing physical vapor deposited (PVD) and electrochemically deposited (ECD) Cu thin films on Si wafers. The study demonstrated that PVD Cu can be bonded at a lower temperature compared to ECD Cu, suggesting the improved bondability may be attributed to significant GB diffusion on the smaller grain size and an increased concentration of random high-angle GBs. Another possible mechanism is surface diffusion. Liu et al.^23,24^ reported that the utilization of ECD highly (111)-oriented Cu polycrystalline thin films, known as nanotwin Cu, improved bondability. They suggested that the improved bondability can be primarily attributed to surface diffusion, as the surface diffusion coefficient of Cu on the (111) plane is three to four orders of magnitude larger than that on other surfaces. Additionally, Shie et al.^21^ attempted to provide a comprehensive bonding mechanism considering both surface and GB diffusion. They assumed a bonding behavior consisting of initial contact formation, GB formation on the contacted surfaces, migration of GBs at the bonding interface, void ripening, and grain growth eliminating the bonding interface. The effect of crystallographic orientation on surface diffusion has been extensively discussed; however, the impact of crystallographic orientation on GB diffusion and its contribution to bondability remain unclear.

Molecular dynamics (MD) simulation, an atomistic-scale simulation method, can be a powerful tool for understanding the bonding behavior and diffusion phenomena at the atomic level. Li et al.^25^ investigated the diffusion phenomenon at the Al–Cu interface using MD simulations and discussed the dominant diffusion mechanism from the perspective of activation energy. Xydou et al.^26^ investigated the void-closing behavior of Cu–Cu GBs and highlighted the importance of GB diffusion. Long et al.^27^ reported the mechanism of microweld formation and the breakage of ultrasonically bonded wires using MD simulations. Thus, MD simulations offer valuable insights into atomic migration behavior. Furthermore, our previous investigation^28^ explored the morphological evolution of the solid-state bonding interface, specifically the Au–Cu interface, by examining atomic diffusion. Through atomic displacement analyses and diffusion coefficient estimations, this examination successfully probed the behavior of interfacial atom penetration into interstices, which was described as interfacial densification behavior. Therefore, our previously established MD simulation and analysis techniques can provide novel perspectives on the bonding behavior of Cu–Cu solid-state bonding, particularly the influence of surface diffusion and GB diffusion on the bonding mechanism.

In this study, MD simulations were employed to elucidate the Cu–Cu solid-state bonding behavior, focusing on interfacial densification and diffusion phenomena. This study aimed to investigate how the crystal orientation of the bonding surfaces affects the bonding behavior. A Cu slab was constructed with a simplified periodic surface structure to simulate surface roughness. The Cu slabs were subsequently bonded at specific temperatures and pressures. The simulation results were analyzed by considering atom displacement and calculating the diffusion coefficient. Based on these results, the bonding behavior in Cu–Cu solid-state bonding was elucidated.

## Methods

**Figure 1:**
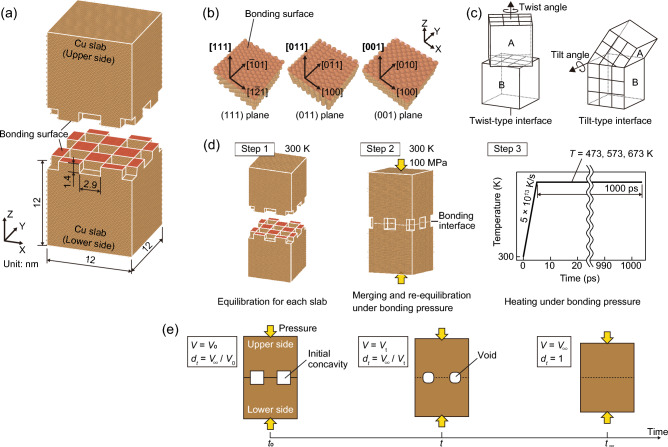
MD simulation setup of Cu–Cu joint models: (**a**) joint model dimensions, (**b**) bonding surface orientations in this study, (**c**) schematic representations of twist- and tilt-type interfaces, (**d**) MD simulation procedure, and (**e**) schematic of relative density evolution.

In this study, MD simulations were conducted using Large-scale Atomic/Molecular Massively Parallel Simulator (LAMMPS) software^29^. To describe the atom interactions, the well-established embedded atomic method (EAM) potential for Cu, as reported by Mishin et al.^30^, was applied. The selected EAM potential accurately represents important properties of Cu, such as its lattice constant, cohesive energy, and elastic modulus^30^. Additionally, the MD-calculated intrinsic (stable) (44.4 mJ/m^2^) and unstable stacking fault energy (158 mJ/m^2^)^30^ closely matched the experimental values of 45 mJ/m^2^^31^ and 162 mJ/m^2^^30^, respectively. The lattice constant and atomic mass of Cu were set to 0.36147 nm and 63.546, respectively. The MD simulation model and procedure are illustrated in Fig. 1. A joint model composed of monocrystalline slabs with identical or different crystal orientations was used, as shown in Fig. 1a. The dimensions of the monocrystalline slab along the X, Y, and Z directions were set to approximately 12 nm. The bonding surfaces were perpendicular to the Z axis. Periodic rectangular concavities and convexities were created on the bonding surfaces of each monocrystalline slab to represent surface roughness. The dimensions of each concave and convex feature were approximately 2.9 × 2.9 × 1.4 nm. The dimensions of the simulation model were adjusted to maintain the periodicity of the atomic arrangement in each monocrystalline slab with a specific crystal orientation, as described in the following paragraph. The total number of atoms in the simulated joints was approximately 250,000.

To evaluate the effect of the crystal orientation of the bonding surfaces on bondability, joint models were constructed using combinations of bonding surfaces, as listed in Table 1. We evaluated the joint models of the {111}, {110}, and {100} bonding surfaces, which are the representative planes of the face-centered cubic (FCC) structure of Cu, as shown in Fig. 1b. The {111}-bonding surface model was created by iterating a 26 × 45 × 19 unit cell fabricated with the [$1\overline{2 }1$], [$\overline{1 }01$], and [$111$] directions aligned with the X-, Y-, and Z-axes. The {110}-bonding surface model was created by iterating a 32 × 45 × 45 unit cell fabricated with the [$100$], [$0\overline{1 }1$], and [$011$] directions aligned with the X-, Y-, and Z-axes. The {100}-bonding surface model was created by iterating a 32 × 32 × 32 unit cell fabricated with the [$100$], [$010$], and [$001$] directions aligned with the X-, Y-, and Z-axes. The initial simulation box size mismatch between the lower and upper slabs along the X- and Y-axes was considered to be less than 0.6%. These simulation models were prepared using the atomic-scale modeling software ATOMSK^32^.

**Table 1:** Bonding interfaces of the simulated joint models.

No	Bonding surfaces		Note	Bonding interface type
	Lower side	Upper side		
1	{111}	{111}	–	No twist nor tilt
2	{111}	{111}	Twisted by 90.0° along the Z-axis	Twist-type interface
3	{110}	{111}	Tilted by 35.3°	Tilt-type interface
4	{110}	{100}	Tilted by 45.0°	Tilt-type interface
5	{100}	{111}	Tilted by 54.7°	Tilt-type interface

In terms of classifying GBs based on their geometric features, the following types are well-known^33^: tilt GBs, which are tilted with respect to the GB plane; twist GBs, which are twisted with respect to an axis perpendicular to the GB plane, and mixed GBs, which are combinations of tilt and twist GBs. The bonding interfaces were analyzed using an analogy to the crystal GB classification shown in Fig. 1c. The {111}/{111} joint No. 1 is a bonding interface with no twist or tilt; the twisted-{111}/{111} joint No. 2 is a twist bonding interface, and the {110}/{111}, {110}/{100}, and {100}/{111} joints No. 3, 4, and 5 are tilted bonding interfaces with tilt-angles of 35.26°, 45.00°, and 54.74°, respectively.

The initial velocities of the atoms were randomly assigned a Gaussian distribution based on the set temperature. Newton's equations of motion were integrated using the Verlet algorithm with a fixed time step of 1 fs. The simulation procedure is shown in Fig. 1d. The simulation was conducted in the following steps: First, the upper and lower monocrystalline slabs were equilibrated separately at 300 K for 50 ps. During the equilibration of each slab, periodic boundary conditions were applied along the X- and Y-axes, whereas nonperiodic and fixed boundary conditions were set along the Z-axis. The boundary surface along the Z-axis was set sufficiently far from the bonding surface to model it as a surface. The equilibration was performed using a canonical (NVT) ensemble. The potential energy in these slabs relaxed after equilibration. In the second step, the slabs were merged into a joint model, which was equilibrated at 300 K for 50 ps while applying a bonding pressure of 100 MPa along the Z-axis. In the third step, the joint model was heated from 300 K to bonding temperatures of 473, 573, and 673 K at a heating rate of 5 × 10^13^ K/s^34^, while maintaining a bonding pressure of 100 MPa along the Z-axis, and then held for 1000 ps. During the second and third steps, an isobaric-isothermal (NPT) ensemble was used with periodic boundary conditions applied in all directions. The boundary surfaces at both edges along the Z-axis were set sufficiently far from the bonding interface to make the interaction between these edge surfaces negligible. The positions and velocities of the atoms were recorded every 1 ps. Atomic configurations were visualized using the Open Visualization Tool (OVITO)^35^. Five calculations were performed for each simulation condition.

During these MD simulations, the densification behavior, which can be regarded as void closure behavior at the bonding interface, was used as an indicator of the bonding progress. The evolution of the relative density was quantitatively investigated, as shown in Fig. 1e. The relative density (*d*_*t*_) at the simulation time step (*t*) of the simulation box was defined as follows:

1 $${d}_{t}=\frac{m}{{V}_{t}}/\frac{m}{{V}_{\infty }}=\frac{{V}_{\infty }}{{V}_{t}}$$

where *m* is the mass in this model, which is constant. *V*_t_ is the simulation box volume at a time step of *t*. *V*_∞_ is the simulation box volume without voids at the bonding interface. Thus, the relative density evolution can be determined from the volume change of the simulation box due to void closure. In addition, void volume, *V*_void,*t*_, is determined as follows:

2 $${V}_{{\text{void}}, t}={V}_{ t}-{V}_{\infty }=(1-{d}_{t}){V}_{ t}$$

To quantitatively evaluate the atomic displacement, the mean square displacement (MSD) of the atoms constituting the bonding interface in each joint was evaluated. MSD was calculated as follows:

3 $$MSD=\langle {\left|{\varvec{r}}\left(t\right)-{\varvec{r}}(0)\right|}^{2}\rangle =\frac{1}{N}\sum_{i=1}^{N}\left({\left|{{\varvec{r}}}_{i}\left(t\right)-{{\varvec{r}}}_{i}(0)\right|}^{2}\right)$$

where *N* is the number of atoms, and ${{\varvec{r}}}_{i}\left(t\right)$ and ${{\varvec{r}}}_{i}(0)$ are the position vectors of atom *i* at times *t* and 0, respectively. The diffusion coefficients (*D*) at the bonding interfaces of each joint were calculated from the MSD results. The diffusion coefficients were evaluated using Einstein's diffusion law^36^.

4 $$D=\underset{t\to \infty }{{\text{lim}}}\frac{1}{2\widetilde{N}t}\langle {\left|{\varvec{r}}\left(t\right)-{\varvec{r}}(0)\right|}^{2}\rangle $$

where $\widetilde{N}$ = 3 denotes system dimensionality. The diffusion coefficient can be estimated as one-sixth of the slope of the linear approximation of the MSD evolution curve. Five calculated results were obtained for each simulation condition.

## Results

### Morphology evolution at bonding interface

**Figure 2:**
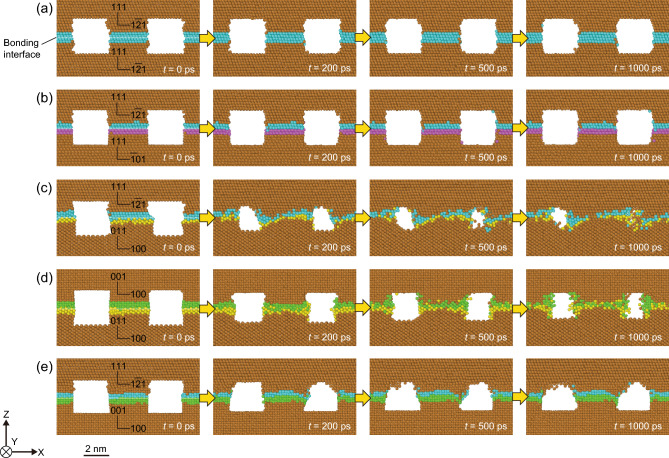
Evolution of the bonding interface morphology of the Cu–Cu joints at 673 K at timesteps of 0, 200, 500, and 1000 ps: (**a**) {111}/{111} joint, (**b**) twisted-{111}/{111} joint, (**c**) {110}/{111} joint, (**d**) {110}/{100} joint, and (**e**) {100}/{111} joint. Atoms arranged at the bonding interface are colored according to their orientation.

First, the evolution of the bonding interface morphology of the Cu–Cu joints was evaluated at 673 K for various bonding surfaces at time steps of 0, 200, 500, and 1000 ps, as shown in Fig. 2. Figure 2a–e show the results for the {111}/{111}, twisted-{111}/{111}, {110}/{111}, {110}/{100}, and {100}/{111} joints. During these MD simulations, the degree of densification at the bonding interface was considered an indicator of the bonding progress. At a time step of 0 ps, the initially arranged concave and convex features remained in all cases, indicating that the equilibration process at 300 K under a bonding pressure of 100 MPa (step 2 in Fig. 1d) did not promote interfacial densification. After heating, the {111}/{111} and twisted-{111}/{111} joints exhibited little densification at the time step of 1000 ps, as shown in Fig. 2a,b, respectively. The arrangement of atoms at the bonding interface changed slightly during the simulation period. In contrast, the {110}/{111}, {110}/{100}, and {100}/{111} joints exhibited densification at the bonding interface, as shown in Fig. 2c–e. The arrangement of the atoms at the bonding interface changed during the simulation. These atoms tended to migrate into the cavities, leading to densification. These results suggest that the misorientation of the bonding interface has a significant effect on the bonding behavior.

**Figure 3:**
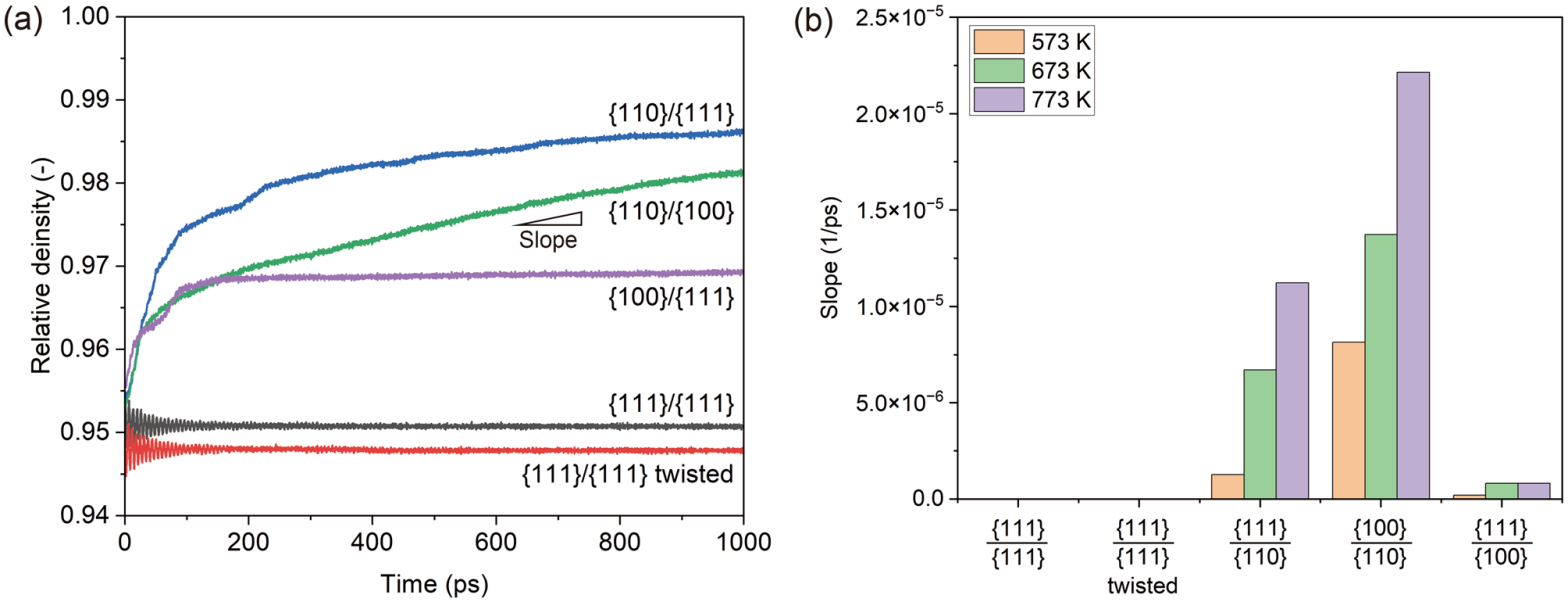
Evolution of relative density for the Cu–Cu joints: (**a**) relationship between simulation time and relative density at 673 K, and (**b**) the slope of the linear approximation of the curves of each joint at 573, 673, and 773 K.

Subsequently, the evolution of relative density at the bonding interface was quantitatively investigated, as shown in Fig. 3. Figure 3a shows the relative density as a function of simulation time at 673 K. Under all conditions, a significant change in the relative density occurred from 0 to 200 ps. During this initial stage (0–200 ps), the {111}/{111} joints exhibited a minor increase in relative density with fluctuations as the system searched for a stable structure, whereas the other joints showed a significant increase. Following the initial stage, a gradual increase in density was observed, particularly for the {110}/{111} and {110}/{100} joints. Notably, this gradual densification process in the later stages is essential for understanding the actual joining process. Therefore, the slope of the linear approximation in Fig. 3a, which indicates the densification rate, was calculated as shown in Fig. 3b. The results indicate that the slopes increased with increasing temperature, that is, densification progressed faster. Additionally, these slopes depended on the orientation relationships of the bonding interfaces. The results suggest that {110}/{100} and {110}/{111} joints accelerate densification at the bonding interface. Thus, the interfacial morphological evolution accompanying densification highlights the significance of the orientation relationship between the bonding surfaces.

### Evolution of local crystal structure

**Figure 4:**
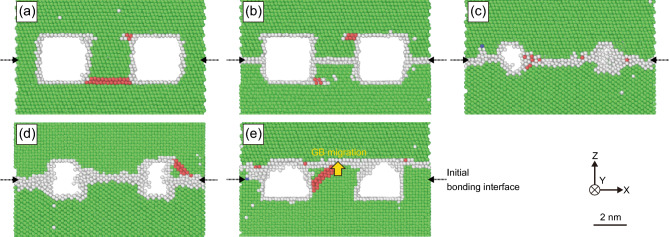
Snapshots of the crystalline structure at the time step of 1000 ps at 673 K for the Cu–Cu joints: (**a**) {111}/{111} joint, (**b**) twisted-{111}/{111} joint, (**c**) {110}/{111} joint, (**d**) {110}/{100} joint, and (**e**) {100}/{111} joint. Atom colors represent the crystalline structures: green for FCC, red for HCP, and gray for amorphous. The initial bonding interfaces are indicated by arrows.

To investigate the bonding behavior at the bonding interface in detail, polyhedral template matching (PTM)^37^, which allows the identification of the local crystalline structure, was used to observe the evolution of the atomic-scale structure at the bonding interfaces. Figure 4 shows snapshots of the crystalline structure at a time step of 1000 ps at 673 K for each Cu–Cu joint, where green, red, and gray atoms represent FCC, hexagonal close-packed (HCP), and amorphous structures, respectively. An amorphous structure was observed on the free surfaces or GBs. In FCC structures, the two-layered HCP atoms can be regarded as stacking faults. In the case of the {111}/{111} joint with no twisting or tilting, as shown in Fig. 4a, no crystalline structure misarrangement was observed at the original bonding interface, indicating that the joint interface was a single crystal. In the other cases, an amorphous structure, shown in gray, was observed at the bonding interface, indicating that these joint interfaces can be considered as GBs. In the cases of the twisted or tilted joints shown in Fig. 4b–d, the bonding interface remained at the initial location. Contrastingly, only the {100}/{111} joint, as shown in Fig. 4e, exhibited GB migration to the edge of the voids on the upper {111} side. Furthermore, stacking faults were observed in the vicinity of all interfaces, as indicated by the red atoms. These results indicate that the combination of crystal orientations constituting the bonding interface caused differences in the local interface structure.

### Atomic diffusion behavior

**Figure 5:**
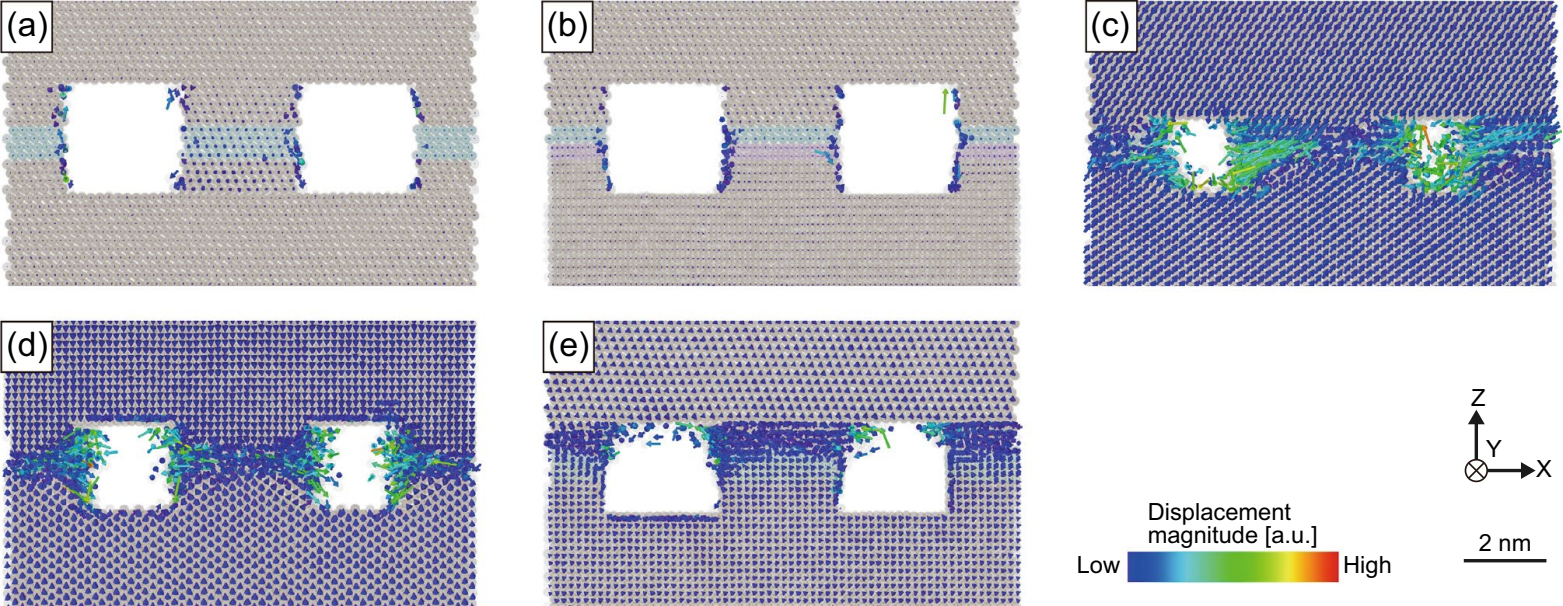
Atomic displacement behavior at the time step of 1000 ps at 673 K for the Cu–Cu joints: (**a**) {111}/{111} joint, (**b**) twisted-{111}/{111} joint, (**c**) {110}/{111} joint, (**d**) {110}/{100} joint, and (**e**) {100}/{111} joint. Atoms are shown translucently.

Atomic displacement vector analysis was performed to elucidate the atomic diffusion behavior related to interfacial densification, as shown in Fig. 5. First, a small atomic displacement was observed in the {111}/{111} joints, regardless of whether they were twisted, as shown in Fig. 5a,b. Additionally, atom diffusion on the void surfaces, called surface diffusion, occurred in only a small amount. In contrast, joints with different crystalline orientations at the bonding interface, as shown in Fig. 5c–e, exhibited significant atom diffusion on the X–Y plane toward the voids. Based on the locations of the interfaces shown in Fig. 4, these significant atom diffusions are considered GB diffusion. These results indicate that the GB diffusion in the bonding interfaces with different orientations was significant, whereas the surface diffusion remained minimal.

Comprehensive analyses of the morphological evolution, local crystalline structure, and atomic displacement at the bonding interfaces revealed that the bonding process predominantly involved GB diffusion formed by the two contacting bonding surfaces, with minimal contribution from surface diffusion. Moreover, this process was affected by the type of crystalline misorientation. In the following section, the diffusion phenomena caused by misorientation are discussed using quantitative analyses.

## Discussion

As demonstrated by MD simulations that involved five different joint models, the crystalline orientations of the bonding surfaces play a critical role in the bonding process. We observed that the bonding interface densified more slowly when the crystalline orientations were identical and significantly faster with mismatched orientations. The misoriented bonding surfaces provided GB structures at the interface and significant atomic displacements at these boundaries. This suggests a potentially pivotal role of GB diffusion in the bonding progression.

**Figure 6:**
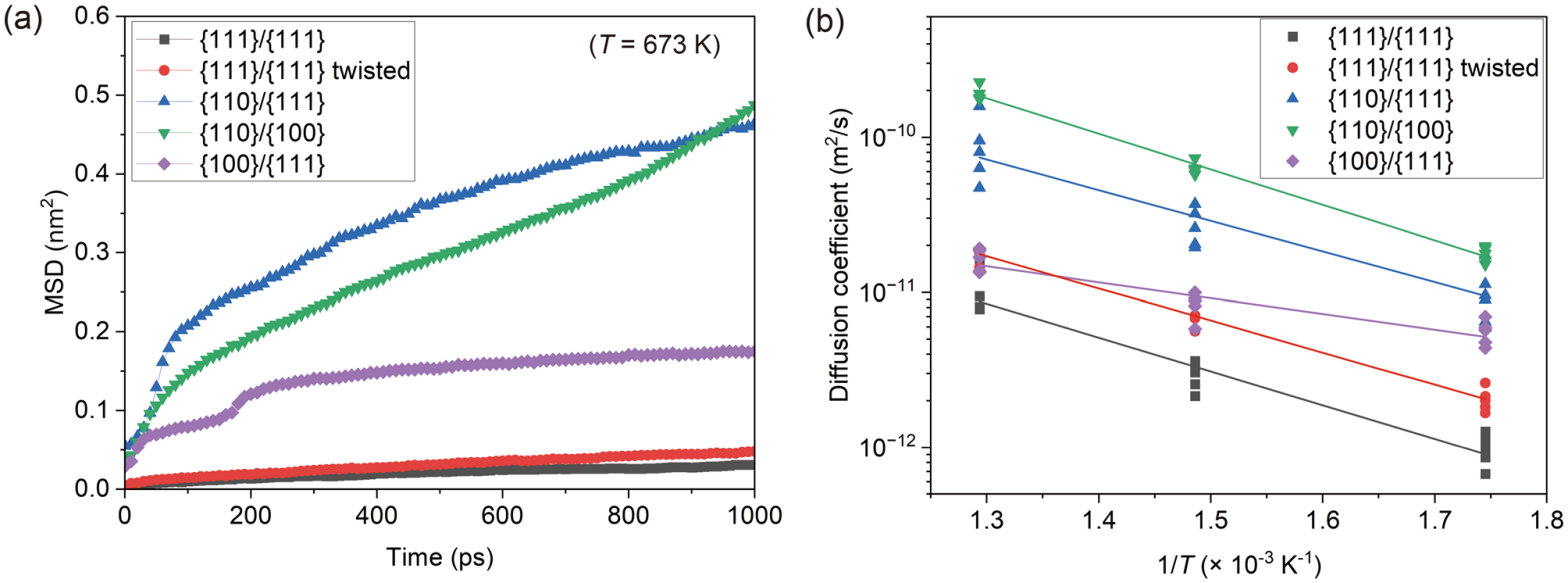
(**a**) MSD evolution of atoms constituting the bonding interface in each joint at 673 K. (**b**) Arrhenius plot of diffusion coefficients calculated using the atoms constituting the bonding interface in each joint at different temperatures, providing the activation energy and frequency factor, as shown in Table 2.

We investigated the diffusion coefficients calculated using the MSD and compared them with those obtained in other studies to further discuss the diffusion mechanism. Atoms with a thickness of 1 nm along the Z-axis at the bonding interface were utilized for the MSD calculations. These atoms correspond to those constituting the GB at the bonding interface at a time step of 1000 ps, as shown in Fig. 4. The MSDs calculated at 673 K are shown in Fig. 6a. This shows that in the {111}/{111} joints, the MSD was small regardless of the presence of a twist, and its rate of increase over time was minimal. In contrast, the MSDs calculated for the {110}/{111}, {110}/{100}, and {100}/{111} joints were significantly larger than those observed in the {111}/{111} joints.

**Table 2:** Activation energies ($Q$) and frequency factors (${D}_{0}$) calculated from the Arrhenius plots shown in Fig. 6b.

Configuration	$Q$(eV)	${D}_{0}$(m^2^/s)	$D$ at 573 K (m^2^/s)	Refs
{111}/{111}	0.43	5.7 × 10^–9^	9.0 × 10^–13^	This work
{111}/{111} twisted	0.41	8.4 × 10^–9^	2.1 × 10^–12^	This work
{110}/{111}	0.39	2.7 × 10^–8^	9.5 × 10^–12^	This work
{110}/{100}	0.46	1.7 × 10^–7^	1.7 × 10^–11^	This work
{100}/{111}	0.20	3.1 × 10^–10^	5.2 × 10^–12^	This work
Lattice diffusion	2.19	7.8 × 10^–5^	4.6 × 10^–24^	^38^
GB diffusion*	0.88	2.4 × 10^–6^	4.5 × 10^–14^	^39^
Surface diffusion (100)	0.38	1.2 × 10^–7^	5.5 × 10^–11^	^40^
Surface diffusion (110) ||	0.23	8.0 × 10^–8^	7.6 × 10^–10^	^40^
Surface diffusion (110)⊥	0.30	3.2 × 10^–6^	7.4 × 10^–9^	^40^
Surface diffusion (111)	0.026	3.0 × 10^–8^	1.8 × 10^–8^	^40^

*Grain boundary width was calculated as 0.5 nm.

Based on the MSD calculation results, the diffusion coefficients (*D*) at the bonding interfaces of each joint at varying temperatures of 573, 673, and 773 K were calculated. The results were compiled into Arrhenius plots, as shown in Fig. 6b. Five plots were constructed for each condition. Across all joints, a linear relationship between the logarithm of the diffusion coefficient and the inverse of the temperature (1/*T*) was observed. The {111}/{111} joints with and without twisting exhibited comparably small diffusion coefficients. In contrast, the misoriented joints had substantially larger diffusion coefficients than their homogeneous counterparts. This implies that bonding interfaces with different crystal orientations potentiate GB diffusion.

Furthermore, the slope of the Arrhenius plots provides insight into the activation energy ($Q$) and frequency factor (${D}_{0}$), as per the following formula:

5 $$D={D}_{0}\mathrm\,{exp}(-\frac{Q}{RT})$$

6 $${\text{ln}}\left(D\right)=-\frac{Q}{R}\cdot \frac{1}{T}+{\text{ln}}\,({D}_{0})$$

where *R* denotes the gas constant. The calculated $Q$ and ${D}_{0}$ values are listed in Table 2. The $Q$ in this study ranged from 0.20 to 0.46 eV, which is significantly lower than the value for lattice diffusion (2.19 eV) and even smaller than that for typical GB diffusion (0.88 eV). They are close to the value for surface diffusion (0.23–0.38 eV), except for the (111) surface. Focusing on the diffusion coefficient at 573 K, the values obtained in this study were approximately 10^–12^ m^2^/s, which is smaller than the typical range of surface diffusion values (10^–11^–10^–8^ m^2^/s) and two orders of magnitude larger than the typical value for GB diffusion (10^–14^ m^2^/s).

The atomic displacement in this study clearly occurred at the GB, as shown in Fig. 5. However, it is interesting to note that the activation energy in this study was lower than that previously reported for GB diffusion using randomly oriented polycrystals. This may be attributed to the crystallographic orientation relationship of the GB diffusion behavior. The consistency of the atomic arrangement at GBs is widely known to affect their stability. The effect of misorientation on the GB stability has been systematically discussed for twist^41^ and symmetric tilt GBs^42^ with considering relative tangential translations between the two surfaces; however, this is outside the scope of this study. This explains how the instability of the GBs serves as the driving force behind the transport and rearrangement of atoms around them. The calculated potential energy of the atoms constituting the GBs in the misoriented joints followed the order of ${E}_{{\text{p}}}^{\left\{110\right\}/\{100\}}>{E}_{{\text{p}}}^{\left\{110\right\}/\{111\}}>{E}_{{\text{p}}}^{\left\{100\right\}/\{111\}}$. In other words, the larger the potential energy possessed by the atoms at the GBs, the larger the diffusion coefficient tended to be. Another reason is the effect of several nm-sized voids at the bonding interface. Xydou et al.^26^ investigated the effect of the nm-sized voids placed in GBs on their diffusion behavior and noted the importance of void size for the GB diffusion coefficient and activation energy. When the size of the voids is miniaturized to a few nanometers, the GB diffusion coefficient becomes large, and its activation energy decreases, especially at low temperatures. It is possible that the presence of nm-sized voids near GBs creates instability by free surfaces, which in turn may enhance the mobility of atoms. This explains the larger GB diffusion coefficients observed in this study compared to typical values, as well as the smaller activation energies.

## Conclusion

The comprehensive findings of our study underscore the critical influence of the crystalline orientation of the bonding surfaces on the bonding process. Using MD simulations, we demonstrated that the densification rate of the bonding interface was slower when the orientations were identical but significantly increased when the orientations were misaligned. This effect can be attributed to the GB structures formed at the bonding interface owing to misorientation, implying a possible key role for GB diffusion in bonding progression. The diffusion coefficients calculated using MSD confirmed this finding. The diffusion coefficients were significantly larger for joints with misoriented crystalline orientations than for joints with identical orientations. This further reinforces the hypothesis that the presence of different crystal orientations at the bonding interface fosters GB diffusion. Interestingly, the activation energy for GB diffusion obtained from our simulations was lower than that typically associated with GB diffusion. This deviation can be explained by the influence of the crystallographic orientation relationship on the GB diffusion behavior and the impact of voids at the bonding interface. In conclusion, our research elucidates the role of crystalline orientation in diffusion phenomena at bonding interfaces. These findings provide valuable insights for optimizing the manufacturing processes involving bonding. However, further research is required to fully understand the atomic rearrangements at GBs and their effect on the stability of bonding interfaces in real polycrystalline structures.

## Data Availability

All data generated or analyzed in this study are included in this published article or are available from the corresponding author upon reasonable request.
